# Gaps in Survey Data on Cancer in American Indian and Alaska Native Populations: Examination of US Population Surveys, 1960–2010

**DOI:** 10.5888/pcd10.120258

**Published:** 2013-03-21

**Authors:** Shinobu Watanabe-Galloway, Tinka Duran, Jim P. Stimpson, Corey Smith

**Affiliations:** Author Affiliations: Tinka Duran, Corey Smith, Great Plains Tribal Chairmen’s Health Board, Rapid City, South Dakota; Jim P. Stimpson, University of Nebraska Medical Center, Omaha, Nebraska.

## Abstract

**Introduction:**

Population-based data are essential for quantifying the problems and measuring the progress made by comprehensive cancer control programs. However, cancer information specific to the American Indian/Alaska Native (AI/AN) population is not readily available. We identified major population-based surveys conducted in the United States that contain questions related to cancer, documented the AI/AN sample size in these surveys, and identified gaps in the types of cancer-related information these surveys collect.

**Methods:**

We conducted an Internet query of US Department of Health and Human Services agency websites and a Medline search to identify population-based surveys conducted in the United States from 1960 through 2010 that contained information about cancer. We used a data extraction form to collect information about the purpose, sample size, data collection methods, and type of information covered in the surveys.

**Results:**

Seventeen survey sources met the inclusion criteria. Information on access to and use of cancer treatment, follow-up care, and barriers to receiving timely and quality care was not consistently collected. Estimates specific to the AI/AN population were often lacking because of inadequate AI/AN sample size. For example, 9 national surveys reviewed reported an AI/AN sample size smaller than 500, and 10 had an AI/AN sample percentage less than 1.5%.

**Conclusion:**

Continued efforts are needed to increase the overall number of AI/AN participants in these surveys, improve the quality of information on racial/ethnic background, and collect more information on treatment and survivorship.

## Introduction

The interplay of complex individual and social factors has resulted in cancer- related disparities, including higher cancer incidence and deaths among American Indian and Alaska Natives (AI/ANs) (1). Substantial geographic differences in cancer deaths exist among AI/ANs (2). AIs in the Northern Plains region have a higher incidence of cancer than AI/ANs in other regions and the US population. Among the US AI/AN population, Northern Plains AIs have the highest cancer death rate and one of the highest cancer incidence rates (3).

Comprehensive cancer control (CCC) programs are pooling community resources to reduce the cancer burden through risk reduction, early detection, better treatment, and enhanced survivorship (4). Population-based data are essential for establishing the baselines and measuring the progress made by these programs. The Centers for Disease Control and Prevention (CDC) funds 50 states, 7 tribes or tribal organizations, and 7 US-affiliated Pacific Islands or territories to establish CCC coalitions; assess the burden of cancer; determine priorities; and develop, implement, and evaluate CCC plans (4). However, tribal and territorial CCC programs often experience challenges finding public health information that is specific to their population groups, relevant to their community priorities, or both. 

To increase the understanding of cancer data gaps, we systematically reviewed survey data related to cancer and the AI/AN population. The 3 objectives of this study were to 1) identify major population-based surveys conducted in the United States that contain questions related to cancer, 2) document the sample size of the AI/AN population in these surveys, and 3) identify gaps in the types of cancer-related information collected by these surveys.

## Methods

### Data sources

We identified major survey data sources by conducting an Internet query of US Department of Health and Human Services agency websites. Websites included Agency for Healthcare Research and Quality, CDC, Centers for Medicare and Medicaid Services, Indian Health Service, and the National Institutes of Health, specifically, the National Cancer Institute. We searched for information about data collection systems sponsored by these agencies and reviewed their reports to identify data sources related to cancer. We then conducted a Medline search to identify English-language articles using the terms “cancer” and “survey,” which resulted in more than 10,000 records. We narrowed the search by specifying the author address to include “USA.” On the basis of authors’ knowledge of population-based surveys, most population-based surveys with national or close-to-national coverage were expected to be done sometime after 1960, which was confirmed with the results of the search. We reviewed the abstracts of approximately 5,000 articles to identify additional surveys that were not identified through the Internet search. Most articles reported results from a research study that used a survey as the method for data collection. Some articles reported results from national surveys that were already identified in the website query. The Medline search helped us identify 2 additional sources for potential inclusion: The Health and Retirement Study (HRS) (5) and the National Mortality Followback Survey (NMFS) (6). Although many data collection activities occur at the local or state level, this analysis focused on gaps in existing data with wide geographic coverage.

### Selection of surveys

Surveys were subject to further analysis using 3 inclusion criteria. First, the survey data had to include information from at least 1 of the following categories: cancer risk or protective factors (eg, smoking, obesity, physical activity, vaccination for human papillomavirus), cancer incidence and death, or cancer screening and treatment use. Second, the survey had to be population-based. That is, the purpose of the data collection was to produce a representative sample of the defined target population. If, for example, data collection was conducted on the basis of a convenience sample of patients seen at 1 health clinic, the survey would not meet this inclusion criterion. Third, the survey was required to have national coverage or be based on a national initiative to collect multistate data.

### Data extraction

The unit of analysis was a survey. A data extraction form was created in Microsoft Excel (Microsoft Corporation, Redmond, Washington) to collect detailed information about each survey, including purpose and description, type of cancer information collected, race and tribal affiliation, sample size, data collection methods, and period of data collection. We sought sample size information for the most recently available data at the time of data extraction. To determine the type of information collected by the surveys, we used the continuum of cancer care model used by CCC programs because we wanted to examine the data sources from the perspective of cancer prevention and control to understand their usefulness and limitations. CCC programs include activities across the continuum of cancer care to encourage people to live a healthy lifestyle (prevention), promote cancer screening tests (early detection), increase access to good cancer care (treatment), and improve the quality of life for people who survive cancer (survivorship) (4). To extract information for these 4 phases or categories, we reviewed survey forms and manuals published on the websites of the organizations that administer the surveys. We reviewed all the available forms and manuals across multiple years of data collection.

## Results

### Sampling and data collection methods

Because many of the surveys reviewed target specific groups such as students and new mothers, it is important to be aware of differences in age distribution between the US AI/AN population and the US population. According to the 2010 US census, the median age of the US population was 35.8 years for both men and women, and the median age of the AI/AN population was 29.4 years for men and 31.0 years for women. In 2010, 24% of the US population and 30% of the AI/AN population were younger than 18 years (7).

The American Indian Adult Tobacco Survey (AI-ATS) (8), the Alaska Native Adult Tobacco Survey (AN-ATS) (9), and the South Dakota Tribal Pregnancy Risk Assessment Monitoring System (SD-Tribal PRAMS) (10) are all cross-sectional surveys that sample exclusively from AI/AN communities (Table 1). These surveys provide tribe- and community-specific data. The AI-ATS and AN-ATS handbooks provide detailed information about different sampling methods so that individual tribes and communities can choose the method best suited to their need. 

**Table 1:** Purpose, Race Reporting, Source Population, Methods, and Data Period of National Surveys or Surveys With a National Initiative Conducted in the United States From 1960 Through 2010 that Contain Cancer-Related Information on American Indians/Alaska Natives

Data Title (Sponsor)	Purpose/Description	Race Reporting	Source Population, Methods, and Data Period
American Indian Adult Tobacco Survey (CDC)	To collect tribe- and community-specific data on tobacco use for American Indian populations	American Indian tribal membership or similar list compiled by each community	The manual encourages census sampling for small tribes and random sampling for larger tribes Face-to-face survey The project was funded in 2000 and the operation manual developed
Alaska Native Adult Tobacco Survey (CDC)	To collect tribe- and community-specific data on tobacco use for Alaska Native populations	Alaska Native tribal membership or household list compiled by each community	If lists of people are available, a systematic sampling to produce a proportionate sample. If such lists are not available, area sampling is recommended to do a stratified 2-stage cluster sample of housing units Face-to-face survey The project was funded in 2000 and the operation manual developed
Behavioral Risk Factor Surveillance System (CDC)	To collect uniform, state-specific data on preventive health practices and risk behaviors in the adult population	Self-reported	Adults from all 50 states, the District of Columbia, Puerto Rico, the US Virgin Islands, and Guam Cross-sectional, telephone interview Annual (1984–present)
Health Behavior in School-aged Children (National Institute of Child Health and Human Development/Health Resources and Services Administration)	A cross-national study conducted by the World Health Organization to evaluate well-being, health behaviors, and social context	Self-reported	School-aged children aged 11, 13, and 15 years Cross-sectional, school-based survey with data collected through self-completion questionnaires Every 4 years (1983/1984–present)
Health Information National Trends Survey (National Cancer Institute Division of Cancer Control and Population Sciences)	To collect nationally representative data about the American public’s use of cancer-related information	Self-reported	Adults selected from a list of addresses from the US Postal Service administrative records Cross-sectional, self-administered survey/interview Multiple data collection methods (computer-assisted telephone interview, paper-pencil, web-based) Every 2 years (2003–present)
Health and Retirement Study (National Institute on Aging)	To collect information about physical and mental health, insurance coverage, financial status, family support systems, and retirement planning	Self-reported	Americans older than 50 years Longitudinal survey Face-to-face (first mode), telephone follow-up Annual (1992–1996), every 2 years (1998–2008)
Medical Expenditure Panel Survey — Household Component (Agency for Healthcare Research and Quality and NCHS)	To collect information on health conditions, health status, use of medical services, charges and source of payments, access to care, satisfaction with care, health insurance coverage, income, and employment	Self-reported	A sample drawn from a nationally representative subsample of households that participated in the prior year’s National Health Interview Survey Panel survey Computer-assisted telephone interview Ongoing (1996–present)
National Ambulatory Medical Care Survey (NCHS)	To collect information about the provision and use of ambulatory medical care services in the United States	Reported by health care facility	Nonfederally employed, office-based physicians Cross-sectional Physician or staff sample patients and complete a reporting form for each sampled visit Annual (1973–1981; 1985; 1989–present)
National Health Interview Survey (NCHS)	A household survey that serves as the primary source of information on the health of the US noninstitutionalized, civilian populations	Self-reported	Civilian noninstitutionalized populations residing in the 50 states and the District of Columbia Cross-sectional Personal household interview with computer-assisted personal interviewing Annual (1963–present)
National Health and Nutrition Examination Survey (CDC)	To assess the health and nutritional status of US adults and children; used to determine the prevalence of major diseases and risk factors for diseases	Self-reported	Complex multistage sampling design to obtain a probability sample of the civilian noninstitutionalized US population Audio computer-assisted self-interviewing, computer-assisted personal interviewing, and laboratory tests Program began in the early 1960s; in 1999, the survey became a continuous (annual) program
National Hospital Ambulatory Medical Care Survey (NCHS)	To collect data on the use and provision of ambulatory care services in the emergency and outpatient departments of noninstitutional, general, and short-stay hospitals	Reported by health care facility	Nonfederal general and short-stay hospitals in the 50 states and the District of Columbia Physician or staff sample patients, complete a reporting form for each sampled visit Annual (1992–present)
National Immunization Survey (CDC)	To measure progress toward Healthy People 2010 goals on adolescent vaccinations; 2 components: household survey and provider survey	Self-reported	Random-digit dialing to select households with children Telephone survey of parents (with permission, a mailed survey to vaccination provider) Annual (1995–present)
National Mortality Followback Survey (NCHS)	Uses death certificate data; information provided by proxy to study the etiology of disease, demographic trends in death, and other health issues	Data reported by physicians and other people on death certificates	A sample of US residents who died in a given year Proxy respondent questionnaire completed through a telephone or in-person interview The 1993 survey is the sixth in a series of surveys first initiated in the early 1960s.
National Youth Tobacco Survey (CDC)	To enhance the capacity of states to collect tobacco-use–related information for youths that is not available from other sources	Self-reported	Students in grades 6–12 Cross-sectional School-based survey completed by students in their classrooms; data available for 1999, 2000, 2002, 2004, 2006, 2009, and 2011
South Dakota Tribal Pregnancy Risk Assessment Monitoring System (CDC)	To collect state-specific, population-based data on maternal attitudes and experiences before, during, and shortly after pregnancy	Information reported on the birth certificate	Each participating state draws a stratified systematic sample of 100 to 250 new mothers every month from a frame of eligible birth certificates; many states stratify by mother’s race/ethnicity Mothers’ responses linked to extracted birth certificate data items Mailed self-administered survey (telephone follow-up interview if the mailed survey not returned) Annual (1988–present)
Tobacco Use Supplement to the Current Population Survey (National Cancer Institute and CDC)	A key source of national and state-level data on smoking and other tobacco use in the US household population	Self-reported	Civilian, noninstitutionalized population aged 15 years or older Cross-sectional Telephone, in-person interviews Administered as part of the Current Population Survey in 1992–1993, 1995–1996, 1998–1999, 2000, 2001–2002, 2003, 2006–2007
Youth Risk Behavior Survey (CDC)	To monitor the prevalence of risk behaviors that most influence health in youths; to identify factors that contribute to unintentional injuries and violence	Self-reported	National survey includes nationally representative sample of public and private high school students School-based survey completed by students in their classrooms Every 2 years (1991–2011)

Abbreviations: CDC, Centers for Disease Control and Prevention; NCHS, National Center for Health Statistics.

Three surveys (Health Behavior in School-aged Children [HBSC], the National Youth Tobacco Survey [NYTS], and the Youth Risk Behavior Survey [YRBS]) are school-based, cross-sectional surveys that target children and adolescents. The HBSC was the only cross-national survey reviewed in this study. All 3 surveys collect data on smoking and other risk behaviors among children and youth. The Tobacco Use Supplement to the Current Population Survey (TUS-CPS) (11) is a cross-sectional telephone survey of smoking and other tobacco uses among people aged 15 years or older. The TUS-CPS produces state-specific data and a nationally representative sample by using a multistage stratified sample of households based on US census data. The National Immunization Survey (NIS) (12) also focuses on children. The NIS is a cross-sectional survey that collects information about vaccination history among infants and young children (aged 19–35 months) and teens (aged 13–17 years). Unlike data collected in the HBSC, NYTS, and YRBS, the NIS data are collected from parents and vaccination providers.

The Behavioral Risk Factor Surveillance System (BRFSS) (13), the National Health Interview Survey (NHIS) (14), and the National Health and Nutrition Examination Survey (NHANES) (15) are among the most comprehensive cross-sectional health surveys conducted in the United States. The BRFSS focuses exclusively on adults, and the NHIS and the NHANES include children. The NHIS and NHANES, which conducts laboratory tests, are 2 of the longest-running surveys sponsored by the National Center for Health Statistics. The Medical Expenditure Panel Survey (MEPS) (16) is an ongoing panel survey that collects information on health conditions, use of and satisfaction with medical services, and medical cost. The survey sample is from a nationally representative subsample of households that participated in the prior year’s NHIS.

The Health Information National Trends Survey (HINTS) (17) is a cross-sectional survey that collects nationally representative data about the public’s use of cancer-related information. The first HINTS survey was conducted in 2003, making it one of the newest population-based surveys on cancer.

The NMFS (6) is a cross-sectional survey that uses death certificate data and information provided by proxy to study the etiology of disease and demographic trends in mortality. The HRS (5) is a longitudinal survey that focuses on issues among adults aged 51 years or older.

Cross-sectional surveys of ambulatory medical care — the National Ambulatory Medical Care Survey (NAMCS) (18) and the National Hospital Ambulatory Medical Care Survey (NHAMCS) (19) — annually collect data on the provision and use of ambulatory medical care services. The NAMCS focuses on office-based physicians, and the NHAMCS focuses on general and short-stay hospitals.

### Race, tribal affiliation, and AI/AN sample size

Most of the data sources reviewed collect general population data from people of different racial/ethnic groups. The exceptions are the AI-ATS, the AN-ATS, and the SD-Tribal PRAMS, which primarily focus on AI/ANs. Eleven surveys collect race/ethnicity information from the survey respondents, 3 (NAMCS, NHAMCS, NMFS) rely on race/ethnicity information found in health care facility administrative data, 2 (AI-ATS, AN-ATS) use tribal membership information provided by the participating tribes, and 1 (SD-Tribal PRAMS) uses race/ethnicity reported in birth certificate data provided by a parent. Tribal affiliation information is found in only 1 data source reviewed in this study: SD-Tribal PRAMS.

Of the 15 surveys for which sample size information was available (AI-ATS and AN-ATS are not included because the data are owned by participating tribes that require approval to access the information), only 6 had an AI/AN sample size larger than 500: BRFSS, NHIS, TUS-CPS, NYTS, SD-Tribal PRAMS, and HBSC (Table 2). Three surveys reported an AI/AN sample size smaller than 100: HINTS (n = 61), NHANES (n = 86), and NAMCS (n = 93). The percentage of AI/AN respondents in the total sample ranged from 0.4% for NAMCS to 8.5% for NYTS. In the US 2000 census, 1.5% of people self-identified as AI/AN; only 4 surveys (NYTS, HBSC, YRBS, BRFSS) exceeded a percentage higher than 1.5%. Currently, 4 surveys (HINTS, HRS, NHIS, and NHANES) oversample minorities, but none of them oversample the AI/AN population.

**Table 2:** Sample Size of Total Respondents and AI/AN Respondents of National Surveys or Surveys with a National Initiative Conducted in the United States From 1960 Through 2010 that Contain Cancer-Related Information^a^

Data Source (Data Collection Year)^b^	Total Sample Size	AI/AN Sample Size (% of Total Sample)
Behavioral Risk Factor Surveillance System (2008)	414,509	6,470 (1.6)
Health Behavior in School-aged Children (2005–06)	9,016	526 (5.8)
Health Information National Trends Survey (2007)	7,674	61 (0.8)
Health and Retirement Study (1992 Core)	12,521	162 (1.3)
Medical Expenditure Panel Survey (2006)	32,577	275 (0.8)
National Ambulatory Medical Care Survey (2004)	25,286	93 (0.4)
National Health Interview Survey (2008)	291,893	3,192 (1.1)
National Health and Nutrition Examination Survey (2007–08)	10,149	86 (0.8)
National Hospital Ambulatory Medical Care Survey (2004 Outpatient)	31,783	149 (0.5)
National Immunization Survey (2008 Household)	30,681	396 (1.3)
National Mortality Followback Survey (1993)	22,957	205 (0.9)
National Youth Tobacco Survey (2011)	18,866	1,596 (8.5)
South Dakota Tribal Pregnancy Risk Assessment Monitoring System (2007)	NA	948 (NA)
Tobacco Use Supplement to the Current Population Survey (2008–09)	237,199	1,851 (0.8)
Youth Risk Behavior Survey (2007)	14,041	297 (2.1)

Abbreviation: AI/AN, American Indian/Alaska Native; NA, not available.

a The American Indian Adult Tobacco Survey (AI-ATS) and Alaska Native Adult Tobacco Survey (AN-ATS) are not included in this summary because the data are owned by participating tribes that require approval to access the information.

b The date in parentheses is the data collection year of the sample size information presented in this table. We sought to obtain sample size information for the most recently available data for each survey at the time of data extraction.

### Cancer information collected

A total of 15 surveys reviewed in this project contain information about cancer risk and protective factors (Table 3), primarily health behaviors or lifestyle factors. Use of commercial tobacco (ie, tobacco products available to the general public) is addressed by 14 surveys, but the AI-ATS is the only survey that collects data on use of sacred tobacco (ie, traditional tobacco used by American Indians for ceremonial purposes). Physical activity data are collected in 8 surveys, and nutrition information is obtained by 6 surveys. Other less commonly collected information includes tobacco policies (AI-ATS, AN-ATS, BRFSS, TUS-CPS), sexual behavior (NHANES, YRBS), occupational exposure (NHIS, NMFS), and household pesticide use (BRFSS, NHANES).

**Table 3:** Cancer Information Domain Coverage of National Surveys or Surveys with a National Initiative Conducted in the United States From 1960 Through 2010 that Contain Cancer-Related Information on American Indians/Alaska Natives **a**

Domain or Topic^b^	No. of Surveys That Covered Domain/Topic
**Prevention**	
**Tobacco and alcohol**	
Commercial tobacco use	14
Sacred tobacco use	1
Secondary smoke exposure	7
Smoking cessation	9
Tobacco policies	4
Tobacco advertising and access for youths	2
Alcohol use	6
**Physical activity, nutrition, and obesity**	
Physical activity	8
Nutrition	6
Obesity (body mass index)	8
**Vaccine, infection, and sexual behavior**	
Receipt of human papillomavirus vaccine	4
Receipt of hepatitis B vaccine	4
Human papillomavirus infection	2
Sexual behavior	2
**Other **	
Ultraviolet exposure	4
Occupational exposure	2
Household pesticide use	2
Family history of cancer	2
Knowledge and perception of cancer risk	1
**Early Detection**	
**Screening**	
Receipt of breast cancer screening test	5
Receipt of cervical cancer screening test	5
Receipt of prostate cancer screening test	5
Receipt of colorectal cancer screening test	5
Type of cervical cancer screening tests offered and Pap test guideline used at facility	2
**Diagnosis **	
Self-reported diagnosis	7
Diagnosis based on information collected at health care facilities	1
Availability of diagnostic tests at health care facility	1
**Treatment**	
Receipt of cancer-related treatment	4
Availability of cancer treatment at health care facility	1
Insurance coverage of cancer treatment	2
Insurance denial due to cancer	1
Cost of treatment	1
Participation in clinical trial	2
Complementary and alternative medicine use	2
Patient and provider communication	1
Treatment summary or care plans	1
**Survivorship**	
Activities of daily living, functional disability	5
Limitations caused by cancer	2
Pain and pain control	5
Fatigue	3
Psychological distress	6
Cancer worry and fear of recurrence	1
Social support	3
Cancer care giving experience	1

Abbreviation: Pap, Papanicolaou.

a Surveys were the following: American Indian Adult Tobacco Survey, Alaska Native Adult Tobacco Survey, Behavioral Risk Factor Surveillance System, Health Behavior in School-aged Children, Health Information National Trends Survey, Health and Retirement Study, Medical Expenditure Panel Survey, National Ambulatory Medical Care Survey, National Health Interview Survey, National Health and Nutrition Examination Survey, National Hospital Ambulatory Medical Care Survey, National Immunization Survey, National Mortality Followback Survey, National Youth Tobacco Survey, South Dakota Tribal Pregnancy Risk Assessment Monitoring System, Tobacco Use Supplement to the Current Population Survey, and Youth Risk Behavior Survey.

b Cancer domains or topics presented in this table are based on information found in the survey forms and survey manuals published on the websites of the organizations that administer the surveys. We reviewed all the available forms across multiple years.

Nine surveys obtain information about cancer screening tests or diagnosis. Five surveys (BRFSS, HINTS, HRS, MEPS, NHIS) provide information about the receipt of cancer screening tests based on self-reported data. Two surveys (NAMCS, NHAMCS) collect information from health care providers to determine types of cervical cancer screening tests offered at the facility. These surveys also collect information on the Pap test guideline used at the facility. Seven surveys (BRFSS, HINTS, HRS, MEPS, NHANES, NHIS, NIS) collect self-reported cancer diagnosis information. NAMCS collects information about availability of cancer diagnostic tests at a given health care facility.

Treatment and survivorship information data are less frequently collected than data on prevention and early detection. Only 7 surveys obtain treatment information. Receipt of cancer-related information is the most commonly covered topic (HRS, MEPS, NHIS, NHANES). Less commonly collected data include complementary and alternative medicine use (HINTS, MEPS), participation in clinical trials (BRFSS, NHIS), cost of treatment (MEPS), and patient and provider communication (HINTS). Only 6 surveys cover topics related to cancer survivorship. More commonly collected survivorship information includes psychological distress (BRFSS, HINTS, HRS, MEPS, NHIS, NHANES), disability (BRFSS, HRS, MEPS, NHIS, NHANES), pain (BRFSS, HRS, MEPS, NHIS, NHANES), and fatigue (HRS, MEPS, NHIS).

## Discussion

To our knowledge, this is the first review to identify the limitations and strengths of cancer survey data sources for the AI/AN population. Our review identified strengths and limitations of existing population-based surveys that obtain cancer-related information. Many surveys date before the 1990s and provide data that can be used to look at trends in cancer risks, protective behaviors, incidence, and outcomes. Many of the surveys reviewed in this study provide data for the Healthy People program to establish national objectives and track progress in cancer and other health issues. However, estimates specific to the AI/AN population are often lacking because of inadequate AI/AN sample size.

Because approximately 1.5% of the US population identify as AI/AN, oversampling is required to produce a representative sample of AI/AN respondents. However, achieving an adequate AI/AN sample size is challenging, even with oversampling. For instance, some surveys, such as NHANES, have a limited total sample size and are administered in selected geographic areas each year. In addition, oversampling may not have a substantial effect on AI/AN sample sizes for facility-based surveys such as the NAMCS and the NHAMCS, which exclude federally employed physicians and federal hospitals. Another option, which may be used alone or in conjunction with oversampling, is to use poststratification weighting to ensure that AIs are adequately represented in survey samples. 

Nevertheless, the findings that 9 national surveys reviewed in this study had an AI/AN sample size smaller than 500 and that 10 had an AI/AN sample percentage smaller than 1.5% were surprising. The current practice in designing and implementing survey data collection for race and ethnicity needs to be rethought. For instance, the sample size of surveys that are used to develop health indicators should be large enough to produce AI/AN-specific estimates. Some questions are age- and sex-specific (eg, colorectal and breast cancer screening use) and require an even larger sample of AI/AN respondents to produce reliable estimates after stratification. Conducting the surveys less frequently could offset the cost of oversampling.

We identified surveys that collect a range of cancer-related data, from prevention to survivorship. In terms of prevention, smoking, physical activity, and nutrition are addressed by many surveys. NHANES is unique because self-reported measures of cancer risk can be validated by laboratory test data. HINTS, a newer survey, collects data on cancer information communication. These types of data can be useful for examining variations in information use and communication style across different population groups. In general, surveys reviewed in this study put much greater emphasis on risk factors and screening use than on treatment and survivorship. More people are living with cancer and surviving longer. Population-based data on access to and use of cancer treatment and follow-up care as well as barriers to receiving timely and quality care should be collected more consistently. For AI/ANs, who have a lower cancer survival rate than some other racial/ethnic groups (20), this information is especially important. Because of the complexity of cancer therapy, medical records and claims data may be a better source of information on treatment than surveys. Medical records and claims data also provide comorbidity and cost information. The work of the Office of the National Coordinator for Health Information Technology on the meaningful use of data (21) will increase the usability of clinical data and can supplement information collected from surveys.

This study has limitations. This review was designed to inform federal policy and practice for collection of chronic disease surveillance data. Therefore, we focused on major national health surveys. The decision to restrict our analysis to the examination of population health data meant that potentially relevant data from research studies may have been overlooked. Also, we did not examine other cancer data sources such as registries and administrative databases that supplement treatment information, which is often missing in survey data. Future research may examine issues unique to these data sources.

The Affordable Care Act, Section 4302, will address health inequalities by improving collection of data on race and ethnicity, sex, and primary language (22). The implementation of new data standards began in 2012, and the new survey questions will be standard on most surveys sponsored by the US Department of Health and Human Services (23). The revised survey question for race will continue to list AI/AN separately. The new survey questions may improve the quality of data on race and ethnicity, which could lead to more accurate estimates of all racial/ethnic categories. These improvements may have the added benefit of increased rates for the AI/AN population. 

## References

[1] American Cancer Society. Cancer facts and figures 2012. Atlanta (GA): American Cancer Society; 2012. http://www.cancer.org/acs/groups/content/@epidemiologysurveilance/documents/document/acspc-031941.pdf. Accessed July 15, 2012.

[2] Espey D , Paisano R , Cobb N . Regional patterns and trends in cancer mortality among American Indians and Alaska Natives, 1990–2001. Cancer 2005;103(5):1045–53. 10.1002/cncr.20876 15685622

[3] Espey DK , Wiggins CL , Jim MA , Miller BA , Johnson CJ , Becker TM . Methods for improving cancer surveillance data in American Indian and Alaska Native populations. Cancer 2008;113(5):1120–30. 10.1002/cncr.23724 18720372

[4] National Comprehensive Cancer Control Program. Centers for Disease Control and Prevention. http://www.cdc.gov/cancer/ncccp/. Accessed July 15, 2012.

[5] Health and Retirement Study. University of Michigan. http://hrsonline.isr.umich.edu/. Accessed September 5, 2012.

[6] National Mortality Followback Survey. Centers for Disease Control and Prevention. http://www.cdc.gov/nchs/nvss/nmfs.htm. Accessed September 5, 2012.

[7] American fact finder 2012. US Census Bureau; 2012. http://factfinder2.census.gov/faces/nav/jsf/pages/searchresults.xhtml?refresh=t. Accessed October 17, 2012.

[8] American Indian Adult Tobacco Survey Work Group. American Indian Adult Tobacco Survey implementation manual. Atlanta (GA): US Department of Health and Human Services, Centers for Disease Control and Prevention; 2008.

[9] Albright VA , Mirza S , Caraballo R , Niare A , Thorne SL . Guidance document for administrating the Alaska Native Adult Tobacco Survey. Atlanta (GA): US Department of Health and Human Services, Centers for Disease Control and Prevention; 2010.

[10] South Dakota Tribal Pregnancy Risk Assessment Monitoring System. Northern Plains Tribal Epidemiology Center. http://www.aatchb.org/nptec/mch.php?page=2. Accessed September 5, 2012.

[11] Risk factor monitoring and methods. National Cancer Institute. http://riskfactor.cancer.gov/ . Accessed September 5, 2012.

[12] National Immunization Survey. Centers for Disease Control and Prevention. http://www.cdc.gov/nchs/nis.htm. Accessed September 5, 2012.

[13] Behavioral Risk Factor Surveillance System. Centers for Disease Control and Prevention. http://www.cdc.gov/brfss/index.htm. Accessed September 5, 2012.

[14] National Health Interview Survey. Centers for Disease Control and Prevention. http://www.cdc.gov/nchs/nhis.htm. Accessed September 5, 2012.

[R15] National Health and Nutrition Examination Survey. Centers for Disease Control and Prevention. http://www.cdc.gov/nchs/nhanes.htm. Accessed September 5, 2012. 15771149

[16] Medical Expenditure Panel Survey. Agency for Healthcare Research and Quality. http://meps.ahrq.gov/mepsweb/. Accessed September 5, 2012. 38416859

[17] Health Information National Trends Survey. National Cancer Institute. http://hints.cancer.gov/. Accessed September 5, 2012.

[18] Ambulatory Health Care Survey. Centers for Disease Control and Prevention. http://www.cdc.gov/nchs/ahcd.htm. Accessed September 5, 2012.

[19] Kunen S , Prejean C , Gladney B , Harper D , Mandry CV . Disposition of emergency department patients with psychiatric comorbidity: results from the 2004 National Hospital Ambulatory Medical Care Survey. Emerg Med J 2006;23(4):274–5. 10.1136/emj.2005.027367 16549572 PMC2579500

[20] Jemal A , Siegel R , Xu J , Ward E . Cancer statistics, 2010. CA Cancer J Clin 2010;60(5):277–300. 10.3322/caac.20073 20610543

[21] Health IT Policy Committee. http://www.healthit.gov/policy-researchers-implementers/federal-advisory-committees-facas/meaningful-use. Accessed January 28, 2013.

[22] Reducing health disparities with improved data collection. Office of Minority Health. http://minorityhealth.hhs.gov/templates/content.aspx?ID=9232&lvl=2&lvlID=208. Accessed September 5, 2012.

[23] Data collection standards for race, ethnicity, sex, primary language, and disability status. Office of Minority Health. http://minorityhealth.hhs.gov/templates/content.aspx?lvl=2&lvlid=208&id=9227#Race. Accessed September 5, 2012. 10.1001/jama.2011.1789 22147383

